# Unexpected TBEV Seropositivity in Serbian Patients Who Recovered from Viral Meningitis and Encephalitis

**DOI:** 10.3390/pathogens11030371

**Published:** 2022-03-17

**Authors:** Pavle Banović, Adrian Alberto Díaz-Sánchez, Selena Đurić, Siniša Sević, Vesna Turkulov, Dajana Lendak, Sandra Stefan Mikić, Verica Simin, Dragana Mijatović, Ivana Bogdan, Aleksandar Potkonjak, Sara Savić, Dasiel Obregón, Alejandro Cabezas-Cruz

**Affiliations:** 1 Ambulance for Lyme Borreliosis and Other Tick-Borne Diseases, Department of Prevention of Rabies and Other Infectious Diseases, Pasteur Institute Novi Sad, 21000 Novi Sad, Serbia; draganav77@gmail.com; 2 Department of Microbiology with Parasitology and Immunology, Faculty of Medicine in Novi Sad, University of Novi Sad, 21000 Novi Sad, Serbia; 3 Department of Biology, University of Saskatchewan, 112 Science Place, Saskatoon, SK S7N 5E2, Canada; adiasanz88@gmail.com; 4 Faculty of Medicine in Novi Sad, University of Novi Sad, 21000 Novi Sad, Serbia; djuric.selena@gmail.com (S.Đ.); sinisa.sevic@mf.uns.ac.rs (S.S.); vesna.turkulov@mf.uns.ac.rs (V.T.); dajana.lendak@mf.uns.ac.rs (D.L.); sandra.stefan-mikic@mf.uns.ac.rs (S.S.M.); 5 Clinic for Infectious Diseases, Clinical Center of Vojvodina, 21000 Novi Sad, Serbia; 6 Department of Microbiology, Pasteur Institute Novi Sad, 21000 Novi Sad, Serbia; luketic.s@mts.rs (V.S.); ivana.basaric@gmail.com (I.B.); 7 Department of Veterinary Medicine, Faculty of Agriculture, University of Novi Sad, 21000 Novi Sad, Serbia; ale@polj.uns.ac.rs; 8 Scientific Veterinary Institute “Novi Sad”, 21000 Novi Sad, Serbia; sara@niv.ns.ac.rs; 9 School of Environmental Sciences, University of Guelph, Guelph, ON N1G 2W1, Canada; dasielogv@gmail.com; 10 Anses, INRAE, Ecole Nationale Vétérinaire d’Alfort, UMR BIPAR, Laboratoire de Santé Animale, F-94700 Maisons-Alfort, France

**Keywords:** tick-borne encephalitis, seroreactivity, human encephalitis, *Ixodes ricinus*

## Abstract

The tick-borne encephalitis virus (TBEV) causes a life-threatening disease named Tick-borne encephalitis (TBE). The clinical symptoms associated with TBE range from non-specific to severe inflammation of the central nervous system and are very similar to the clinical presentation of other viral meningitis/encephalitis. In consequence, TBE is often misclassified by clinical physicians, mainly in the non-identified high-risk areas where none or only a few TBE cases have been reported. Considering this situation, we hypothesized that among persons from northern Serbia who recovered from viral meningitis or encephalitis, there would be evidence of TBEV infection. To test this hypothesis, in this observational study, we evaluated the seroreactivity against TBEV antigens in patients from northern Serbia who were hospitalized due to viral meningitis and/or viral encephalitis of unknown etiology. Three cases of seroreactivity to TBEV antigens were discovered among convalescent patients who recovered from viral meningitis and/or encephalitis and accepted to participate in the study (*n* = 15). The clinical and laboratory findings of these patients overlap with that of seronegative convalescent patients. Although TBE has been a notifiable disease in Serbia since 2004, there is no active TBE surveillance program for the serologic or molecular screening of TBEV infection in humans in the country. This study highlights the necessity to increase the awareness of TBE among physicians and perform active and systematic screening of TBEV antibodies among patients with viral meningitis and/or encephalitis.

## 1. Introduction

Tick-borne encephalitis (TBE) is one of the most important zoonotic viral tick-borne diseases in Europe and Asia [1]. The causative agent, tick-borne encephalitis virus (TBEV), is a neurotropic positive-strand RNA virus of the genus *Flavivirus*, family Flaviviridae [2]. To date, five TBEV subtypes have been described based on phylogenetic clustering and geographical distribution, although different subtypes can be found in each endemic region in different proportions. TBEV subtypes include Western (European; TBEV-Eu), Siberian (Eastern; TBEV-Sib), Far-Eastern (TBEV-Fe), Baikalian (TBEV-Bkl), and Himalayan (TBEV-Him) [3,4,5]. In addition to their geographical distribution, these five subtypes also differ in clinical presentation [6]. The TBEV is mainly transmitted to humans by ticks, which act as vectors and reservoirs simultaneously. Occasionally, TBEV can be transmitted to humans gastrointestinally via unpasteurized milk products from infected goats and sheep [7,8]. Although several tick species have been shown to be competent vectors for the transmission of TBEV throughout Eurasia, *Ixodes ricinus* is the most important tick species related to human TBE cases in Western and Central Europe, as well as in the Balkan region [7,8,9,10,11].

During the last decades, TBE has become a major public health concern in Europe due to the geographical expansion of TBEV, the extended season for transmission, and the mortality rates ranging from 0.5–2% for TBEV-Eu up to 35% for TBEV-Fe [12]. Although the course of TBEV infection is in most cases asymptomatic or with only mild flu-like illness, the virus can also cause severe inflammation of the central nervous system (CNS) [13,14]. Particularly, the full manifestation of disease caused by TBEV-Eu has a characteristic biphasic course. In the initial viremic phase, patients show nonspecific febrile illness associated with headaches, myalgias, fatigue, and muscle and joint pain [7]. This phase usually lasts less than a week, and once the viremia decreases, the patient improves for a few days before the second phase [15]. However, after this short asymptomatic period, patients enter a second phase characterized by neurological symptoms. Most patients (50–60%) present meningitis, 30–40% meningoencephalitis, and 5–10% meningoencephalomyelitis [7,16,17]. Due to a relatively short period of viremia in TBE patients, TBEV can be detected in patient blood or cerebrospinal fluid (CSF) only during the febrile (initial) phase [18]. Therefore, an essential tool of TBE diagnostics in patients who develop the full clinical picture is the detection of specific anti-TBEV or TBEV-neutralizing antibodies [19].

In general, there is a lack of awareness among practitioners, clinicians, epidemiologists, and virologists of many European countries, including Serbia, about the incidence and clinical forms of TBE [20]. This may be due to several factors. Firstly, medical practitioners working in non-endemic areas will often not raise clinical suspicion toward TBEV infection in patients with viral meningitis/encephalitis. Especially in the cases when patients cannot recall possible tick exposure. Secondly, some countries lack specialized laboratories to detect anti-TBEV or TBEV-neutralizing antibodies (e.g., Serbia) [21]. In Serbia, the first report of TBEV was nearly 50 years ago in a hard tick collected from the Pešter plateau [22]. Several decades later, Potkonjak et al. detected TBEV-Eu subtype in *I. ricinus* collected in the Belgrade area and Fruška Gora Mountain, both in northern Serbia [10]. In addition, Serbian patients infested by *I. ricinus* were seroreactive to TBEV antigens, which suggests possible circulation of TBEV, or other members of the Flaviviridae family, within the Serbian population, as vaccination against TBEV in Serbia is still not widely implemented [23]. Nevertheless, clinical reports of TBE in Serbia remain scarce and underreporting likely exists because of being neglected or misclassified by clinical physicians, mainly in the identified high-risk areas where none or only a few TBE cases have been reported [21,23,24,25]. 

Considering the above precedents, we hypothesized that among persons who recovered from viral meningitis or encephalitis, there would be evidence of TBEV infection. Particularly, in patients living in territories where active TBEV foci have been reported. To test this hypothesis, in the present study, we evaluated the seroreactivity against TBEV antigens in patients from northern Serbia who were hospitalized due to viral meningitis and/or viral encephalitis of unknown etiology.

## 2. Results

### 2.1. Anti-TBEV Seroreactivity among Individuals with Clinical Symptoms of Viral Meningitis and Encephalitis

From a total of 103 persons previously hospitalized in the Clinical Center of Vojvodina due to encephalitis and meningitis, we contacted 95 of them. Of them, 16 patients (16/95; 16.84%) accepted to be enrolled in the study and reported to Pasteur Institute Novi Sad for blood sampling. Inspection of medical records revealed that among enrolled individuals, one had West Nile Virus (WNV) RNA detected in CSF during hospitalization. Accordingly, this patient was removed from the study due to possible cross-reaction of anti-WNV antibodies with TBEV antigens. Anti-TBEV antibodies were detected in 3 of the 15 subjects (3/15; 20%). No enrolled subjects reported previous immunization against TBE or Yellow fever.

### 2.2. Description of TBEV Seroreactive Patients

Case #1

A 47-year-old men was admitted to Clinic for Infectious Diseases on August 16th, 2018, seven days after the disease onset. Main complaints during the disease onset were malaise, pain in the muscles, headache, and fever. No pathological findings were noticed in relation to cardiovascular, hepatic, gastrointestinal, and genitourinary systems. Meningeal signs were negative. Lumbar puncture was performed on the 1st day of hospitalization and CSF findings indicated the development of viral meningitis (Supplementary Table S1). Antiedematous therapy (20% mannitol and dexamethasone) and symptomatic therapy were initiated. In the first several days after hospitalization patient was complaining about disorientation, headache, instability, and dizziness. Testing for WNV (PCR and ELISA IgM/IgG) was negative, after which serological tests for Coxsackievirus and Adenoviral infections were ordered. All tests returned as negative, except for the Coxackie B virus, where border-line reactivity was detected. After 15 days of hospitalization, all complaints were resolved except intermittent headaches and the patient was discharged from the hospital as recovered.

Case #2

A 44-year-old man was hospitalized in the clinical center of Vojvodina on June 2nd, 2018, five days after disease onset. Initial signs and symptoms were headache, neck pain, nausea, chills, and sweating. The patient was firstly examined by a neurologist who ordered computed tomography of the endocranium. As the analysis showed, no abnormal findings patient was transferred to Clinic for Infectious Diseases. The patient denied the existence of fever, photophobia, or vomiting prior to hospitalization. No pathological findings were noticed in relation to cardiovascular, hepatic, gastrointestinal, and genitourinary systems. Meningeal signs were positive. Laboratory findings during hospitalization were without significant deviations from physiological values (Supplementary Table S2). Lumbar puncture was performed on the 1st day of hospitalization and CSF findings indicated the development of viral meningitis (Supplementary Table S2). The patient received parenteral antibiotic therapy (ceftriaxone), antiedematous therapy (20% mannitol and dexamethasone), gastroprotective therapy (pantoprazole), and symptomatic therapy. Control lumbar puncture was performed on the 10th day after hospitalization and CSF findings were within physiological values. On the 11th day of hospitalization, electroencephalography was performed and no significant deviations from physiological values were found. The patient was discharged from hospital 12 days after admission to the Clinic for Infectious Diseases as they recovered.

Case #3

A 12-year-old boy who was recovered from rhombencephalitis in June 2017 was admitted to Clinic for Infectious Diseases for control Magnetic resonance imaging in February 2018 due to complaints about occasional headaches and instability. The patient denied the existence of fever, photophobia, or vomiting prior to hospitalization. No pathological findings were noticed in relation to cardiovascular, hepatic, gastrointestinal, and genitourinary systems. Meningeal signs were positive. Magnetic resonance imaging showed complete regression of lesions on the brain stem described during the previous hospitalization due to rhombencephalitis. The patient was discharged after 2 days with a recommendation for further medical observation.

### 2.3. Demographic Factors Associated with Cases of Meningitis and Encephalitis Related, or Not, to TBEV Exposure

**Figure 1:**
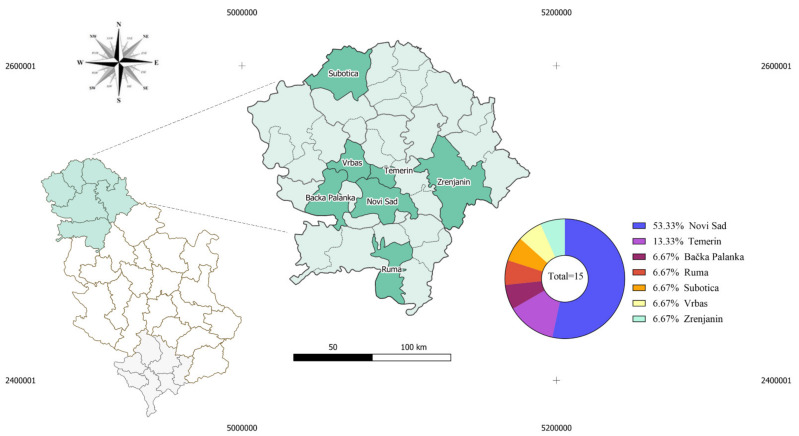
Map of Serbia with the administrative division on NUTS-2 level showing residency municipalities of enrolled patients. Municipalities, where enrolled convalescents live, are marked as green. Municipalities, where TBEV seropositive convalescents were identified, are Novi Sad and Temerin.

Among all enrolled convalescents, Novi Sad city was noted most often as the location of residence (8/15; 53.33%), followed by Temerin town (Temerin municipality) (2/15; 13.33%), Tovariševo village (Bačka Palanka municipality), Ruma town (Ruma municipality), Subotica town (Subotica municipality), Savino village (Vrbas municipality), and Zrenjanin town (Zrenjanin municipality) who were each mentioned once (1/15; 6.66%). Two of the TBEV seroreactive cases were from Novi Sad, while the other was from Temerin (Figure 1). 

From a total of 15 subjects recovered from viral CNS infection, 9 of them were men (9/15; 60%), while 6 were women (6/15; 40%) (Table 1). Chi-square test did not show statistically significant difference in frequency of one gender compared to the other (χ^2^ (1) = 0.2928, *p* = 0.588). The three persons having TBEV antigens reactivity were male (3/3; 100%). Concerning age groups, the majority of convalescents were adults (14/15; 93.33%), and only one subject was in the teenage age group (1/15; 6.66%) (Table 1). Chi-square test showed statistically significant difference in frequency of adult age group over teenagers (χ^2^ (1) = 6.806, *p* = 0.009). In addition, the mean age of enrolled convalescents was found to be approximately 36 years (95% CI: 29.917–41.683). In the TBEV-seroreactive group, two subjects were adults (2/3; 66.66%), while one was a teenager (1/3; 33.33%). The mean age of convalescents reactive with TBEV antigens was 35 years (95% CI: 18–50.6).

Average hospitalization time in all convalescents, regardless of anti-TBEV reactivity, was 11.33 days (95% CI: 8.613–13.987). When anti-TBEV reactivity was taken into account, the average hospitalization period in seroreactive and seronegative was 10 (95% CI: 4.34–15.7) and 11.6 days (95% CI: 8.77–14.4), respectively. No significant difference was found in hospitalization period between TBEV reactive and non-reactive convalescents (*t* = 0.4722; *p* = 0.644).

### 2.4. Clinical and Laboratory Findings in Patients with Anti-TBEV Antibodies

Laboratory results of CSF and blood analyses at the time of hospitalization for the 15 enrolled patients are summarized in Supplementary Tables S1 and S2, respectively. When main complaints at the time of the admission to the hospital were analyzed, we found that the headache (14/15; 93.33%), fever (7/15; 46.66%), vomiting (6/15; 40%) were most common, while tingling sensations and weakness in different body parts (3/15; 20%), disorientation (3/15; 20%), disorganized speech (2/15; 13.33%), fatigue (2/15; 13.33%), short term consciousness loss (1/15; 6.66%), joint pain (1/15; 6.66%), rash (1/15; 6.66%), tinnitus (1/15; 6.66%), and photophobia (1/15; 6.66%) occasionally occurred in the examined cohort. When convalescents were grouped according to TBEV reactivity, we found that headache, fever, and fatigue were only complaints related to TBEV reactive convalescents. More precisely, all TBEV reactive patients reported headache (3/3; 100%), the majority reported fever (2/3; 66.66%), while fatigue was present only in one person (1/3; 33.33%). All of abovementioned complaints were less frequent in TBEV seronegative convalescents (headache - 11/12; 91.66%, fever - 5/12; 41.66% and fatigue - 1/12; 8.33%). Vomiting was the only more common complaint that was not found in the TBEV seroreactive group, although it was present in half of the convalescents from the seronegative group (6/12; 50%) (Table 2).

CSF analysis after lumbar puncture was conducted in all enrolled patients after the admission to the hospital, except for the patient described as Case #3. A common finding in all examined CSF samples acquired from patients with viral meningitis/encephalitis was elevated levels of proteins, as well as increased cell count (hyperproteinorachia and pleocytosis). The CSF cell count and protein levels of Case #1 and Case #2 patients compared to the average levels in TBEV-seronegative patients, were higher and equal, respectively (Table 2). In general, patients with anti-TBEV antibodies had higher protein levels (1.665 g/L; 95% CI: 0.5147–2.8153 g/L) and white blood cell count in CSF (229.5 × 10^6^; 95% CI;1.519–457.481 × 10^6^) when compared to seronegative patients (0.751g/L; 95% CI: 0.61898–0.88302 g/L and 114.3 × 10^6^; 95% CI: 45.718–182.882 × 10^6^, respectively).

## 3. Discussion

Although TBE is considered a neglected disease in many European countries, the incidence of this vaccine-preventable disease in some natural foci has increased in the last two decades, especially in regions where TBEV had not been previously reported [26,27]. This can be attributed to the emergence of new TBEV foci, as well as to the increased awareness of healthcare professionals and upgraded laboratory facilities that allow wide testing of suspected TBE cases [12,28,29].

As the result of long-term surveillance programs in countries traditionally declared as TBE-endemic (e.g., Slovenia, Czech Republic, Austria, Slovakia, among others), we know that the incidence of TBE is showing fluctuations over the years [3,26,30]. The reasons for that are still not fully understood, but higher average temperatures that favor increased tick vector populations and animal reservoirs can be considered as a major driving force of TBEV emergence [31]. Additionally, an increase in the wildlife population favors the migration of ticks to higher altitudes. Warmer weather also allows extended time for people to spend in outdoor activities at green surface areas such as public parks, gardens, and forests, which in turn increase the risk of infected tick bites. 

Since the first TBEV detection in Serbian territory nearly 50 years ago, only locations shown to have active TBEV foci in the recent past are within the Belgrade area and Fruška Gora Mountain [10]. Additionally, Rtanj mountain and near Danube territory in Veliko Gradište municipality were proposed to contain TBEV foci, given that TBEV seroreactive persons acquired tick infestations at those locations [23]. In this study, all seroreactive convalescents were reported to have a residence in municipalities close to Fruška Gora Mountain. 

There were several attempts in recent years to examine possible exposure of the Serbian population to TBEV, mainly via seroprevalence studies [9,10,23,32,33]. According to currently available data, possible exposure to TBEV was in previous years detected only in the population of northern Serbia and the Belgrade area [10,25,32]. More precisely, anti-TBEV antibodies were found in hospitalized patients and blood donors from Novi Sad (seroprevalence 0.37% and 7.9%, respectively). In contrast, no anti-TBEV antibodies were detected in blood donors from the south Serbian region (Nišava district) [9]. The most recent study reported that persons from Serbia previously infested by *I. ricinus* show TBEV seroprevalence of 13.27% [23]. In the absence of effective surveillance programs, this data could indirectly suggest that TBEV circulation within the Serbian population is possibly neglected. Results from this study support the given possibility, as anti-TBEV antibodies were found in 20% (3/15) of examined persons discharged with a diagnosis of viral meningitis or encephalitis. 

Due to the overall small number of participants enrolled in this study, we could not identify specific attributes that could be analyzed as risk factors for TBEV exposure. Parameters such as age and hospitalization period showed no difference when TBEV seropositive convalescents were compared to seronegative ones. Nevertheless, it should be mentioned that all TBEV seropositive convalescents here were men. This finding is not surprising given that men had a 5 times bigger relative risk of being TBEV seropositive after exposure to *I. ricinus* infestation in Serbia [23]. 

TBEV seroreactive patients from Serbia are considerably younger (35 years (95% CI: 18–50.6) when compared to TBE patients from central Europe, where the average age was reported to be 50 years (range 0–88) [34]. The probable reason for this difference is the bias in our study caused by targeted enrollment of persons who recovered from viral CNS infections as persons of older ages have higher possibilities for the development of sequelae and lethal outcomes. It should also be noted that although we found anti-TBEV antibodies in enrolled convalescents, we do not know the specific etiology of their viral CNS infection, as TBEV exposure could happen even after their hospital discharge. On the other hand, the study from central Europe evaluated patients with confirmed TBE diagnosis and inclusion was not dependent on disease outcome (survived/non-survived) [34].

All convalescents with anti-TBEV antibodies enrolled in our study reported to the hospital with signs and symptoms related to meningitis (headache, fever, and fatigue). Meningitis manifested by high fever, headache, nausea, vomiting, and vertigo is the most common clinical form of TBE in central Europe [16]. Nevertheless, none of the TBEV seroreactive convalescents enrolled in this study had problems with vomiting or vertigo.

It should be mentioned that major limitations of this study are the small sample size and bias caused by the selection of the persons who managed to recover from viral CNS infection and were able to visit Pasteur Institute for blood sampling during the coronavirus disease pandemic (survivorship bias). The main value of this dataset is the information about probable exposure to TBEV, while larger-scale studies are needed to show the general frequency of TBEV-related etiology in total cases of viral CNS infections for the Serbian population. The establishment of active surveillance for TBE in Serbia will allow us to form an objective perception of the burden related to TBEV infection in this central Balkan state.

## 4. Materials and Methods

### 4.1. Ethical Declaration

This study was approved by the ethical committee of the Faculty of Medicine in Novi Sad (Ethical approval No 01-39/9/1 from 12 February 2021) and the ethical committee of the Clinical Centre of Vojvodina (Ethical approval No 00-302 from 8 July 2020). All data and material analysis were conducted according to the “Declaration of Helsinki” and “The Patient Rights Law” of the Republic of Serbia.

### 4.2. Study Design and Participant Enrollment

A retrospective study was conducted among persons from northern Serbia who previously recovered from viral meningitis and/or viral encephalitis of unknown etiology to assess their possible exposure to TBEV. Medical history documentation was searched for patients hospitalized at the Infectious Disease Clinic in Novi Sad in the period between 1.1.2018–31.12.2019 and released with one of the following diagnoses: Viral meningitis, Unspecified viral encephalitis, or Unspecified viral infection of the central nervous system. Identified patients were contacted via telephone to introduce the study and ask for their approval to be enrolled. Persons who accepted to participate in the study were asked to report to Pasteur Institute Novi Sad for blood sampling. Informed consent forms were also signed at this stage. After obtaining informed consent, full medical documentation related to each convalescent hospitalization case was assessed and analyzed. All laboratory results and CSF findings acquired at the time of patient hospitalization were collected and processed. All subjects were asked for and medical documentation was crosscheck for previous vaccinations against TBE or Yellow fever.

### 4.3. Detection of Anti-TBEV IgG

For each patient, 3 mL of blood was acquired via phlebotomy in BD Vacutainer^®^ SST™ Tubes (BD, Franklin Lakes, NJ, USA). Blood in the tube was stored at room temperature to clot and serum was separated via centrifugation at 2000× *g* for 10 min. After separation, serum samples were inactivated at 56 °C and finally used for detection of anti-TBEV IgG reactivity via commercial immunofluorescence assay (IFA, Euroimmun, Lübeck, Germany; Cat. No. FI 2661–1005) according to manufacturer instructions with inclusion of positive and negative controls provided with the assay. IFA results were interpreted in qualitative form (i.e., positive or negative), whereas positive finding was noted in specific fluorescence was present in 1:10 serum dilution. Immunofluorescence was analyzed on microscope Leica DM 3000 (Leica, Wetzlar, Germany) with a light source from a mercury bulb using an N2.1 filter (Leica, Wetzlar, Germany) with an excitation wavelength of 515–560 nm.

### 4.4. Data and Statistical Analysis

To identify possible patterns in clinical symptoms or demographic characteristics related to TBEV seroreactivity among encephalitis and meningitis-recovered individuals, we analyzed parameters such as gender, age, residence location, hospitalization length, main complaints during the hospitalization, comorbidities, as well as results of any laboratory analysis related with confirmation of any flaviviral infection during the hospitalization period. Age groups of enrolled subjects were formed considering age ranges as children (1–10 years of age), teenagers (11–20), adults (21–64), and seniors (≥65). Chi-squared test with Yates correction was used for examination of non-parametric variables. Student t-test was used to examine parametric variables between seroreactive and seronegative convalescents. Statistical analyses were performed in GraphPad Prism v.8.0.1 (GraphPad Software Inc., La Jolla, CA, USA). Differences were considered significant when *p* < 0.05. 

## 5. Conclusions

Patients who are reactive with TBEV antigens do not present with clear clinical, demographic or routine laboratory findings compared to seronegative ones. This highlights the necessity for the introduction of TBEV screening tests (e.g., PCR or IgM serology on admission and IgG during hospitalization) in patients who are showing clinical signs of viral CNS infection. Finally, 2–3 weeks after symptom onset, TBEV-neutralizing antibodies should be searched for final diagnosis confirmation, in the case of negative PCR or positive serology finding. Implementation of this diagnostic protocol would aid health practitioners in the process of patient management and allow better outcome prognosis. In addition, better diagnostic procedures will identify endemic TBE risk-areas where vaccination against TBEV should be recommended to reduce the number of infections in Serbia.

## Figures and Tables

**Table 1:** Patient distribution according to gender and age.

Parameter	TBEV Reactive	TBEV Non-Reactive	Total
Gender			
Male	3	6	9
Female	0	6	6
Age			
Children	0	0	0
Teenagers	1	0	1
Adults	2	12	14
Seniors	0	0	0

**Table 2:** Clinical and laboratory findings.

Parameters	TBEV Reactive			TBEV Non-Reactive
	Case#1	Case#2	Case#3	Ave (SD)
Clinical				
Chief complaints	Fatigue, headache, fever	Headache, nausea, fever	Intermittent headaches	N/A*
Comorbidities	None reported	Hyperlipoproteinemia	pyelonephritis	N/A*
Gender	Male	Male	Male	N/A*
Age (years)	43	46	14	36.16 (10.24)
Laboratory (CSF)				
Appearance	Clear	Blurred	N/A	N/A*
Color	Colorless	Light pink	N/A	N/A*
White Blood Cell count (×10^6^)	65	394	N/A	83 (88.72)
Granulocytes (×10^6^)	N/A	18	N/A	41.5 (43.44)
Lymphocytes (×10^6^)	N/A	376	N/A	99.75 (127.91)
Monocytes (×10^6^)	N/A	N/A	N/A	69.5 (48.14)
Glucose level (mmol/L)	N/A	2.9	N/A	3.79 (1.30)
Protein level (g/L)	0.84	2.49	N/A	1.00 (0.81)
Bacterial presence	negative	negative	negative	N/A*

N/A—data not available; N/A*—average value could not be extrapolated due to the nature of the variable.

## Data Availability

Not applicable.

## Supplementary Materials

The following supporting information can be downloaded at: https://www.mdpi.com/article/10.3390/pathogens11030371/s1.
